# Knowledge of and Attitudes toward Suicide and Depression among Japanese in Municipalities with High Suicide Rates

**DOI:** 10.2188/jea.15.48

**Published:** 2005-05-10

**Authors:** Nobuo Nishi, Mie Kurosawa, Masaru Nohara, Shigenori Oguri, Fuminori Chida, Kotaro Otsuka, Akio Sakai, Akira Okayama

**Affiliations:** 1 Department of Hygiene and Preventive Medicine, Iwate Medical University, School of Medicine.; 2 Department of Insurance, Ministry of Health, Labour and Welfare.; 3 Department of Neuropsychiatry, Iwate Medical University, School of Medicine.; 4 Department of Preventive Cardiology, National Cardiovascular Center.

**Keywords:** Suicide, Depression, psychosocial factors, standardized mortality ratio

## Abstract

BACKGROUND: Knowledge of and attitudes toward suicide and depression have not been fully investigated in Japan.

METHODS: Study areas comprised municipalities in northern Japan where standardized mortality ratios (SMRs) from suicide compared with a Japanese standard ranged from 1.62 to 3.72 in men and from 1.43 to 3.49 in women. We conducted a questionnaire survey on a random sample of 7,136 participants aged 20 to 79 years, and analyzed data of 5,547 (77.7%) subjects. We categorized seven municipalities, from which the subjects were drawn, into three groups according to the SMR from suicide. Self-Rating Depression Scale (SDS) score was used for evaluation of depressive states.

RESULTS: The SDS score was significantly higher in the high SMR group in women, but no significant difference among the three SMR groups was observed in men. The percentage of subjects with nine years or less of education was significantly higher in the high SMR group both in men and in women. The percentage of men who drank alcohol once a week or more was significantly higher in the high SMR group. The percentages of subjects unaware that depressive states are treatable by medication were not significantly different among the three SMR groups both in men and in women, while the percentage of men unwilling to see a psychiatrist when depressed was the lowest in the high SMR group.

CONCLUSION: Although a significant difference in SDS score was observed in women, most of the psychosocial factors or knowledge of and attitudes toward suicide and depression were not adversely associated with SMR group.

Japan has one of the highest suicide rates among developed countries.^1^ The number of suicide deaths in Japan increased sharply from 23,494 (18.8 per 100,000 people) in 1997 to 31,755 (25.4 per 100,000 people) in 1998, and it has not decreased since.^2^ This dramatic increase from 1997 through 1998 is Japan’s third peak of suicide mortality since World War II, the first and the second peaks being in 1958 and in 1986, respectively. However, it was the first time that the number of suicide deaths exceeded 30,000. The latest peak is characterized by suicide deaths of middle-aged men and is due to Japan’s prolonged economic recession since the 1990’s.^3^^,^^4^ Suicide prevention is now a major public health challenge in Japan.

Standardized mortality ratios (SMRs) from suicide (the suicide mortality rate in Japan from 1981 through 2000 being used as the standard) in the 47 prefectures of Japan ranged from 0.82 to 1.53 in men and from 0.78 to 1.53 in women.^5^ Aihara and Iki^6^ conducted an ecological study using prefectures of Japan as units of analysis, and found that the proportion of elderly and economic variables such as job application rate are associated with suicide mortality. The SMRs of Iwate Prefecture, on which our study is based, were 1.45 in men and 1.39 in women.^5^ The mortality rate from suicide is particularly high in the northern part of Iwate Prefecture, and the reason for this has been examined by an ecological study using districts as units of analysis.^5^ Among various demographic and socioeconomic variables, it has been shown that unemployment rate for men, and number of hospital beds per 100,000 people and number of doctors per 100,000 people for women were significantly related to the SMR from suicide.

The risk of suicide is high in people with psychological disorders.^7^ Fujita and Kurisu^8^ conducted a study in Japan using the Vital Statistics of Japan and revealed that patients with mental disorders had a four- to five-times higher risk of suicide compared with the general population. Tamakoshi et al.^9^ found in a five-year follow-up study of Japanese middle-aged workers that depressive moods increased the risk of suicide. Takahashi et al.^10^ reported that about 60% of those hospitalized for attempting suicide had mood disorders such as major depression, bipolar depression, or dysthymia. Thus, epidemiologic studies in Japan have revealed an association between depression and suicide, but the extent of awareness among people regarding this association has not been fully investigated, especially in regions with a high suicide rate.

Public knowledge regarding depression and suicide is one of the components of mental health literacy, which is defined as “knowledge and beliefs about mental disorders which aid their recognition, management or prevention.”^11^ In an Irish report in 1991, two-thirds of the nationally representative sample regarded those suffering from depression as either mentally ill or weak willed, and 27% considered antidepressant medication to be ineffective.^12^ In the baseline study of the Defeat Depression Campaign in Great Britain, conducted from 1991 through 1997, the proportion of participants who considered antidepressants to be ineffective for depression was 54%, the number that was reduced to 40% after the six-year intervention.^13^ Although surveys on public attitudes regarding depression have been conducted in cross-sectional studies or in intervention studies, differences in public attitudes with respect to depression exist among areas with different suicide mortality rates.

The aim of this study was to investigate knowledge of and attitudes toward depression and suicide in a large community sample of residents among municipalities with high suicide rates in Japan and to examine relationships between these factors and suicide mortality.

## METHODS

### Questionnaire survey

A questionnaire survey was carried out from February through June in 2002. For the survey, we selected four of six municipalities in the Kuji District, and three of seven municipalities in the Miyako District, which is located south of the Kuji District. Among the seven municipalities selected for the study, three (two in Kuji and one in Miyako) are in coastal areas and four (two in Kuji and two in Miyako) are located inland. A total of 7,136 people, aged 20 to 79 years, were randomly selected from the Basic Resident Register of each of the seven municipalities. Random sampling was done by city workers of the municipalities with the aid of our research members, and in one of the municipalities subjects were randomly selected from those aged 20, 25, 30, 35, 40, 45, 50, 55, 60, 65, 69, 74 and 79 years.

A questionnaire with a letter explaining the objective of the survey was sent to each potential subject. The questionnaire was used to obtain written informed consent for participation from the subjects. Reminder letters were sent to the subjects once or twice. Respondents were offered the choice of a 500-yen gift certificate, a highlighter pen set, or a magnifying glass as a reward for participating. Respondents were requested by mail or telephone to provide any information missing from the questionnaire. As a result of such efforts, we obtained responses from 5,676 subjects (response rate: 79.5%).

Data of 5,547 (77.7%) subjects (2,602 men and 2,945 women) who had missing values in no more than two items of the Self-Rating Depression Scale (SDS) score were used for analyses.^14^^,^^15^ The numbers of samples, the numbers of subjects, and response rates (the numbers of subjects divided by the numbers of samples) by sex and age group (20 to 39, 40 to 59, and 60 to 79) are shown in Table 1. Response rates were higher in the older age groups both in men and in women, and were lower in the Kuji District study area than in the Miyako District study area in all sex and age groups.

**Table 1.** Sample size, number of subjects, and response rate by sex and age group in study areas in Kuji District and Miyako District.

Age group (year)	Men					Women				
	
	Samples		Subjects		Response rate (%)	Samples		Subjects		Response rate (%)
			
	n	%	n	%		n	%	n	%	
Study area in Kuji District										
20-39	522	30.0	329	26.4	63.0	508	27.6	360	25.2	70.9
40-59	712	40.9	515	41.3	72.3	702	38.1	559	39.2	79.6
60-79	506	29.1	404	32.4	79.8	632	34.3	507	35.6	80.2
Total	1,740	100.0	1,248	100.0	71.7	1,842	100.0	1,426	100.0	77.4

Study area in Miyako District										
20-39	381	22.3	250	18.5	65.6	397	21.6	305	20.1	76.8
40-59	685	40.0	538	39.7	78.5	609	33.1	515	33.9	84.6
60-79	646	37.7	566	41.8	87.6	836	45.4	699	46.0	83.6
Total	1,712	100.0	1,354	100.0	79.1	1,842	100.0	1,519	100.0	82.5

Total										
20-39	903	26.2	579	22.3	64.1	905	24.6	665	22.6	73.5
40-59	1,397	40.5	1,053	40.5	75.4	1,311	35.6	1,074	36.5	81.9
60-79	1,152	33.4	970	37.3	84.2	1,468	39.8	1,206	41.0	82.2
Total	3,452	100.0	2,602	100.0	75.4	3,684	100.0	2,945	100.0	79.9

The questionnaire items comprised psychosocial factors, lifestyles, knowledge of and attitudes toward suicide and depression, and the Japanese version of the SDS. For the analyses, prevalence of the following conditions was obtained from the responses to the questionnaire. Psychosocial factors included not married (single, divorced, or widowed), education of nine years or less, very dissatisfied with financial situation, living alone, lacking the chance to visit friends or relatives, having no confidants among friends or relatives when depressed, and never participating in community activities. Lifestyles included currently smoke, drink once a week or more, and sedentary (no regular exercise). Knowledge of suicide and depression included no awareness of suicide mortality rates being higher in the region, no knowledge of depressive states being treatable by medication, no knowledge of the location of a psychiatric hospital, and no awareness of mental health activities by local governments. Attitudes toward suicide and depression included a view of suicide as being permissible (“permissible” or “permissible in certain situations”), unwillingness to see a psychiatrist when depressed, and a view of suicide as being unpreventable by community efforts.

### SMRs of the municipalities

We calculated the SMRs (95% confidence intervals)^16^ from suicide over a period of 19 years (1982 through 2000) in the Kuji District (all six municipalities, including the four municipalities in the study) and the Miyako District (all seven municipalities, including the three municipalities in the study), Iwate Prefecture (Table 2). The following materials were used to calculate the SMRs, with Japan serving as the standard population: (1) sex- and age-specific populations in each municipality were from the population census data for 1980, 1985, 1990, 1995 and 2000,^17^ (2) sex- and age-specific death rates from suicide in Japan from 1982 to 2000 were from the Vital Statistics,^2^ and (3) the numbers of suicide deaths in each municipality from 1982 through 2000 were from the Health and Welfare Statistical Annual of Iwate Prefecture.^18^ The SMRs of all municipalities in the Kuji District were higher than those of all municipalities in the Miyako District, both in men and in women. Based on the SMRs of each municipality, we classified the seven municipalities for the study into three groups; low SMR group, middle SMR group, and high SMR group. We adopted different classifications for men and women.

**Table 2.** Populations in the 2000 census and the total number of deaths and standardized mortality ratios (SMRs) with 95% confidence intervals (CIs) from suicide, from 1982 through 2000 in municipalities of Iwate Prefecture.

	Population in the 2000 census	Suicide deaths 1982-2000	SMR (95% CI)*	SMR group^†^
Men				
Iwate Prefecture	681,238	4,967	1.44 (1.40 - 1.48)	
Kuji District				
All (6 municipalities)	32,758	374	2.24 (2.01 - 2.46)	
Study area (4 municipalities)	28,566	338	2.31 (2.07 - 2.56)	
A	17,311	196	2.30 (1.98 - 2.62)	Middle
B	6,666	58	1.62 (1.20 - 2.03)	Low
C	1,654	38	3.72 (2.53 - 4.90)	High
D	2,935	46	3.13 (2.23 - 4.03)	High

Miyako District				
All (7 municipalities)	50,137	448	1.57 (1.43 - 1.72)	
Study area (3 municipalities)	10,284	149	2.36 (1.98 - 2.74)	
E	2,313	27	2.06 (1.28 - 2.83)	Low
F	6,153	89	2.30 (1.82 - 2.78)	Middle
G	1,818	33	2.91 (1.92 - 3.91)	High

Women				
Iwate Prefecture	734,942	2,604	1.35 (1.30 - 1.40)	
Kuji District				
All (6 municipalities)	36,663	197	2.02 (1.74 - 2.30)	
Study area (4 municipalities)	32,077	173	2.04 (1.74 - 2.35)	
A	19,485	97	1.97 (1.58 - 2.37)	Middle
B	7,511	30	1.43 (0.92 - 1.94)	Low
C	1,728	20	3.49 (1.96 - 5.02)	High
D	3,353	26	2.93 (1.80 - 4.06)	High

Miyako District				
All (7 municipalities)	55,437	193	1.19 (1.02 - 1.36)	
Study area (3 municipalities)	11,146	58	1.60 (1.19 - 2.01)	
E	2,487	13	1.75 (0.80 - 2.70)	Middle
F	6,692	35	1.56 (1.04 - 2.08)	Low
G	1,967	10	1.55 (0.59 - 2.51)	Low

* : SMR is based on suicide mortality of Japanese men and women between 1982 and 2000.

† : All seven municipalities in study areas were categorized into three groups according to the SMR for men and women.

Response rates of the questionnaire survey, calculated for this classification, were 75.6% (646/854) in the low SMR group, 77.3% (1,064/1,377) in the middle SMR group, and 73.1% (892/1,221) in the high SMR group in men, and 82.4% (1,504/1,825) in the low SMR group, 80.1% (867/1,082) in the middle SMR group, and 73.9% (574/777) in the high SMR group in women.

## RESULTS

In Table 3, we compared mean age, age-adjusted SDS score, and age-adjusted prevalence of psychosocial factors and lifestyles among the three groups of municipalities classified on the basis of the SMRs from suicide. The difference in mean ages was marginally significant in men (p=0.066) and significant in women (p<0.001). Mean age was the highest in the low SMR group in women. The difference in mean SDS score was not significant in men but was significant in women (p=0.001). The score was the lowest in the low SMR group and the highest in the high SMR group in women.

**Table 3.** A sex-based comparison of mean age and age-adjusted Self-Rating Depression Scale (SDS) score, and age-adjusted prevalence (%) of psychosocial factors and lifestyles by three groups of municipalities according to the standardized mortality ratio (SMR) from suicide.

	Men					Women					
	
	Total	Low SMR	Middle SMR	High SMR	p*	Total	Low SMR	Middle SMR	High SMR	p*
Age (mean ± standard deviation, year)	51.9±16.0	50.9±15.7	52.7±16.0	51.7±16.0	0.066	52.8±16.1	54.2±16.1	50.5±15.8	52.7±16.1	<0.001
Self-Rating Depression Scale score (mean ± standard error)	38.2±7.6	37.9±0.3	38.6±0.2	38.1±0.3	0.166	40.3±7.9	39.8±0.2	40.6±0.3	41.2±0.3	0.001
Psychosocial factors											
Not married (n=5,399)	26.6	25.2	28.4	25.1	0.182	29.1	29.7	27.6	30.6	0.359
Education of 9 years or less (n=5,493)	46.4	48.2	43.3	48.9	0.015	51.9	51.1	49.9	57.7	<0.001
Very dissatisfied with financial situation (n=5,505)	20.2	23.8	19.3	18.3	0.020	15.9	15.1	16.4	16.4	0.548
Living alone (n=5,514)	8.9	8.8	9.8	7.9	0.378	9.6	10.1	7.3	11.8	0.004
Lacking the chance to visit friends or relatives (n=5,518)	22.3	20.0	24.0	21.9	0.159	15.8	15.5	16.1	16.9	0.668
Having no confidant among friends or relatives when depressed (n=5,518)	14.6	14.1	14.9	14.7	0.879	8.5	8.5	8.1	9.2	0.756
Lacking participation in community activities (n=5,525)	16.1	20.6	16.3	13.0	<0.001	16.3	15.5	17.0	16.8	0.497
Lifestyles											
Currently smoke (n=5,523)	48.8	47.9	49.2	48.2	0.850	8.7	8.2	10.9	6.3	0.004
Drink alcohol once a week or more (n=5,504)	58.8	59.5	55.0	62.8	0.002	13.5	13.1	13.2	14.0	0.913
Sedentary (n=5,483)	57.7	58.0	56.9	58.1	0.843	60.5	59.9	60.9	61.1	0.757

* : P-values for continuous data are by analysis of (co-)variance and those for categorical data are by likelihood ratio test using logistic regression analysis.

Among psychosocial factors, the percentage of those with an education of nine years or less was the highest in the high SMR group both in men and in women. The percentage of those who lived alone was the highest in the high SMR group in women, although no significant difference was observed in men. The percentages of those who were very dissatisfied with their financial situation and those who never participated in community activities were also significantly different in men, but the percentage was the highest in the low SMR group. As for lifestyles, the percentage of those who drank alcohol once a week or more was the highest in the high SMR group in men, although no significant difference was observed in women. The percentage of those who were current smokers was the highest in the middle SMR group in women. These results were not materially changed when district was taken into account in the analyses.

In Table 4, we compared age-adjusted prevalence of knowledge of and attitudes toward suicide and depression among the three groups. As for knowledge of suicide and depression, the percentages of those unaware that the suicide mortality rate is higher in the region were significantly different both in men and in women and the associations were negative: the percentage was higher in the low SMR group and lower in the high SMR group. The percentages of those unaware of the location of a psychiatric hospital were significantly different both in men and in women, but the percentage was the highest in the low SMR group in men and in the high SMR group in women. The percentages of those unaware of mental health activities by local governments were significantly different in men, and the percentage was the highest in the middle SMR group. Apart from the comparison among the three groups, the percentages of men and women who had no knowledge were higher than 75% in terms of both “no awareness that the suicide mortality rate is higher in the region (88.1% in men and 84.5% in women)” and “no knowledge of depressive states being treatable by medication (79.5% in men and 76.3% in women).”

**Table 4.** A sex-based comparison of age-adjusted prevalence (%) of knowledge of and attitudes toward suicide and depression by three groups of municipalities according to the standardized mortality ratio (SMR) from suicide.

	Men					Women				
	
	Total	Low SMR	Middle SMR	High SMR	p*	Total	Low SMR	Middle SMR	High SMR	p*
Knowledge										
No awareness that the suicide mortality rate is higher in the region (n=5,434)	88.1	92.3	87.2	84.5	<0.001	84.5	86.2	85.3	79.2	<0.001
No knowledge of depressive states being treatable by medication (n=5,479)	79.5	81.5	79.0	79.0	0.354	76.3	77.3	74.1	77.4	0.195
No knowledge of the location of a psychiatric hospital (n=5,513)	18.2	21.8	17.9	16.2	0.021	17.3	21.1	6.3	24.3	<0.001
No awareness of mental health activities by local governments (n=5,470)	70.4	69.3	73.6	66.8	0.004	66.8	67.1	65.9	66.6	0.913

Attitudes										
A view of suicide as being permissible (n=5,520)	16.2	15.2	16.1	17.0	0.589	13.0	13.6	9.8	15.8	0.004
Unwillingness to see a psychiatrist when depressed (n=5,515)	59.8	63.8	59.4	57.1	0.019	49.4	50.8	48.6	46.5	0.203
A view of suicide as being unpreventable by community efforts (n=,5547)	63.8	67.3	64.8	60.3	0.017	64.3	63.8	65.7	63.5	0.684

* : P-values are by likelihood ratio test using logistic regression analysis.

As for attitudes toward suicide and depression, the percentage of those with the view that suicide is permissible was the highest in the high SMR group in women, although no significant difference was observed in men. The percentages of those unwilling to see a psychiatrist when depressed and those with the view that suicide is unpreventable by community efforts were significantly different in men, but the percentage was the highest in the low SMR group. Apart from the comparison among the three groups, the percentages of men and women unwilling to see a psychiatrist when depressed were as high as 60% in men and 50% in women. These results were not materially changed when district was taken into account in the analyses.

## DISCUSSION

The characteristics of our study involve the subjects; they make up a large random sample of residents in municipalities with high suicide rates in Japan. We examined psychosocial factors, lifestyles, and knowledge of and attitudes toward suicide and depression. Although a significant difference in SDS score was observed in women, most of the psychosocial factors or knowledge of and attitudes toward suicide and depression were not adversely associated with SMR group. The results shown by Nohara et al.^5^ could explain why SDS scores were significantly different among the three SMR groups only in women. Because women are more affected by factors related to medical services than those related to socioeconomic disadvantage, it seems that depression and suicide are more directly associated in women than in men. Qin et al.^20^ showed similar findings in a time-matched nested case-control study in Denmark, namely that a history of psychiatric hospitalization made the increased suicide risk higher in females than in males, and that socioeconomic variables such as unemployment, retirement, and single marital status were significant risk factors for men after controlling for psychiatric admission. Our survey was cross-sectional, however, and it is therefore possible that higher suicide mortality in a certain area affected the emotions of women more than did lower suicide mortality.

There are three possible reasons for the lack of adverse associations. Firstly, as Nohara et al.^5^ reported, higher rates of suicide in these districts are closely associated with socioeconomic disadvantage in men and with factors related to medical services in women. Compared to such socioeconomic or health-care related factors, knowledge of and attitudes toward suicide and depression might only contribute minimally to differences of suicide mortality in the region. Secondly, although the study was conducted using a large community sample drawn from municipalities with SMRs from suicide ranging from 1.62 to 3.72 in men and from 1.43 to 3.49 in women, the study area might have not been large enough to uncover differences in knowledge of and attitudes toward suicide and depression due to similar social and cultural backgrounds. Thirdly, age range of the subjects might have been too broad. Age-specific analyses revealed that in some items knowledge of and attitudes toward suicide were more alike among the three SMR groups in the same age group than among different age groups in the same SMR group (data not shown). Thus, it might have been difficult to find a common difference across age groups among the three SMR groups in knowledge of and attitudes toward suicide and depression.

As for psychosocial factors and lifestyles, significant differences among the SMR groups were observed in education, financial situation, community participation, and alcohol drinking in men, and in education, living alone, and cigarette smoking in women. When subgroup analyses by three age groups (20-39, 40-59, and 60-79 years) were conducted (data not shown), significant differences were found only in the age group 20-39 years, except for financial situation in men where a significant difference was no longer observed. Lack of consistent differences among age groups made interpretation difficult. Education and unemployment were reported to be associated with suicide.^21^ Our study showed similar results in that the percentage of those with an education of 9 years or less was the highest in the high SMR group, both in men and women. Associations between suicide and such lifestyle factors as cigarette smoking,^22^^,^^23^ alcohol drinking,^24^ and physical activity^25^ were also reported. No clear explanation exists for the lack of difference in smoking prevalence among the three SMR groups in men, but the reason for the highest smoking prevalence observed in the middle SMR group in women might have been because urban areas in Japan, which have higher smoking rates than those of rural areas,^26^ were included in that group. The percentage of those not participating in community activities was the lowest and the percentage of those who drink alcohol once a week or more was the highest in the high SMR group in men. This might have been because community participation and alcohol drinking are closely associated in the rural districts of Japan.

As for knowledge of suicide and depression, significant differences among the SMR groups were observed in three items among men and two items among women. When subgroup analyses by three age groups (20-39, 40-59, and 60-79 years) were conducted (data not shown), significant differences were found only in the age groups 40-59 and 60-79 years for “no awareness that the suicide mortality rate is higher in the region” and in the age group 60-79 years for “no knowledge of the location of a psychiatric hospital” and “no awareness of mental health activities by local governments” in men, and in the age group 40-59 years for “no awareness that the suicide mortality rate is higher in the region” and in all age groups for “no knowledge of the location of a psychiatric hospital” in women. These data indicate that middle-aged or elderly people in the high SMR group, especially men, have sufficient knowledge of suicide and depression, which is contrary to our expectations. When subgroup analyses by age group were conducted for attitudes toward suicide and depression (data not shown), significant differences among the SMR groups were found only in the age groups 20-39 and 40-59 years for “unwillingness to see a psychiatrist when depressed” and in the age group 40-59 years for “a view of suicide being unpreventable by community efforts” in men and in the age group 60-79 years for “a view of suicide as being permissible” in women. The data for men indicated that the percentages were the lowest in the high SMR group, again contrary to our expectations. The lowest percentages observed in the middle SMR group for “a view of suicide as being permissible” and for “no knowledge of the location of a psychiatric hospital” might have been because a psychiatric hospital was located in the municipality for the middle SMR group and women in that municipality tended to regard suicide as not permissible.

As mentioned earlier, in the baseline study of the Defeat Depression Campaign in Great Britain, conducted from 1991 through 1997, the proportion of participants who considered anti-depressants to be ineffective for depression was 54%, a number that was reduced to 40% after the six-year intervention.^13^ In a 1991 Irish report, 27% of the nationally representative sample regarded antidepressants as ineffective.^12^ In our study, more than 70% of the subjects did not know that depressive states are treatable by medication. In addition, half of our sample answered that they were not willing to see a psychiatrist when depressed (59.8% of men and 49.4% of women). Patients in Japan with depressive symptoms tend to visit primary care physicians who are less likely to diagnose depression or prescribe antidepressants.^27^^,^^28^ Efforts should therefore be made to encourage residents to visit psychiatrists. Providing residents with sufficient information on depression might result in an increase in consultations with psychiatrists for depression, since in our study the subjects who knew that depressive states are treatable by medication were significantly more likely to visit a psychiatrist when depressed than those who did not know (25.1% vs. 13.9%).

Psychiatric care is restricted in the region. The municipality with the largest population in each of the two districts, the Kuji District and the Miyako District, has psychiatric hospitals or clinics, but the other municipalities lack even the smaller psychiatric clinics. Knowledge of the location of a psychiatric hospital seems to have been largely affected by the location of subject residence. Provision of psychiatric care cannot be expected to increase immediately, but mental health literacy of the residents could be improved in the short term. We will conduct the first community intervention trial for suicide prevention with a control group in Japan. Our sample was randomly drawn from the intervention and control areas, and the sample size was large enough to detect changes in knowledge about whether depressive states are treatable by medication. The evaluation questionnaire survey is scheduled for 2004, only two years after the baseline survey, due to budget constraints. However, we plan to monitor changes in suicide rates for at least five years both in the intervention area and in the control area.

There may be three limitations in our study. The first limitation is the lower response rate in the younger age groups. We sent reminder letters twice to non-respondents. We sent respondents a gift certificate or gift worth 500 yen. We also asked respondents for missing information. We thus expended considerable effort to improve response rates. The second limitation is that subjects were drawn from discrete age strata in one of the municipalities. This might have affected the age distribution of the subjects. However, we only compared the prevalence of the variables by three age groups, and it is unlikely that the discrete sampling in the one municipality distorted the results. The third limitation is that the municipality with the largest population in the Miyako District was not included in the study. The municipality was characterized by rather low SMRs from suicide: 1.27 in men and 1.12 in women. If knowledge of and attitudes toward suicide and depression in this municipality had been surveyed, a clear contrast between urban and rural areas might have become apparent.

In conclusion, although a significant difference of SDS score was observed in women, most of the psychosocial factors or knowledge of and attitudes toward suicide and depression were not adversely associated with SMR group. It was evident that levels of knowledge about suicide and depression were rather low both in men and in women. Based on the results, we will intervene in these municipalities for suicide prevention.

## References

[1] World Health Organization. World Health Statistics Annual 1997-99. WHO. Geneva, 2000.

[2] Statistics and Information Department, Minister’s Secretariat, Ministry of Health, Labour and Welfare. Vital Statistics of Japan. Health and Welfare Statistics Association. Tokyo (in Japanese).

[3] Lamar J Suicides in Japan reach a record high. BMJ 2000; 321: 528. 10.1136/bmj.321.7260.528 PMC1118433 10968804

[4] Fujita T, Tanihata T, Miura Y Geographic characteristics of the steep increase of suicide deaths since 1998. Kosei No Shihyo 2003; 50(9): 27-34. (in Japanese)

[5] Nohara M, Onoda T, Okayama A Regional accumulation of suicide and its related factors. Kosei No Shihyo 2003; 50 (6): 17-23. (in Japanese)

[6] Aihara H, Iki M An ecological study of the relations between the recent high suicide rates and economic and demographic factors in Japan. J Epidemiol 2003; 13: 56-61. 12587614 10.2188/jea.13.56 PMC9538610

[7] Harris EC, Barraclough B Suicide as an outcome for mental disorders. A meta-analysis. Br J Psychiatry 1997; 170: 205-28. 9229027 10.1192/bjp.170.3.205

[8] Fujita T, Kurisu E Suicide deaths among psychiatric patients--a study based on vital statistics. Nippon Koshu Eisei Zasshi 1992; 39: 858-64. (in Japanese) 1477400

[9] Tamakoshi A, Ohno Y, Yamada T, Aoki K, Hamajima N, Wada M, Depressive mood and suicide among middle-aged workers: findings from a prospective cohort study in Nagoya, Japan. J Epidemiol 2000; 10: 173-8. 10860301 10.2188/jea.10.173

[10] Takahashi Y, Hirasawa H, Koyama K, Asakawa O, Kido M, Onose H, Suicide and aging in Japan: an examination of treated elderly suicide attempters. Int Psychogeriatr 1995; 7: 239-51. 8829430 10.1017/s1041610295002006

[11] Jorm AF Mental health literacy. Public knowledge and beliefs about mental disorders. Br J Psychiatry 2000; 177: 396-401. 11059991 10.1192/bjp.177.5.396

[12] McKeon P, Carrick S Public attitudes to depression: a national survey. Ir J Psychol Med 1991; 8: 116-21.

[13] Paykel ES, Hart D, Priest RG Changes in public attitudes to depression during the Defeat Depression Campaign. Br J Psychiatry 1998; 173: 519-22. 9926082 10.1192/bjp.173.6.519

[14] Chida F, Okayama A, Nishi N, Sakai A Factor analysis of Zung Scale scores in Japanese general population. Psychiatry Clin Neurosci 2004; 58: 420-426. 15298656 10.1111/j.1440-1819.2004.01277.x

[15] Zung WWK A self-rating depression scale. Arch Gen Psychiat 1965; 12: 63-70. 14221692 10.1001/archpsyc.1965.01720310065008

[16] Kahn HA, Sempos CT, Statistical methods in epidemiology, Oxford University Press, New York, 98-102.1989.

[17] Statistics Bureau. Population Census of Japan. Ministry of Public Management, Home Affairs, Posts and Telecommunications (formerly Management and Coordination Agency). 1980, 1985, 1990, 1995, and 2000. (in Japanese)

[18] Department of Health and Welfare of Iwate Prefecture. Annual Health Statistics, Iwate. 1982-2000. (in Japanese)

[19] Dupont WD, Statistical modeling for biomedical researchers: a simple introduction to the analysis of complex data, Cambridge University Press, Cambridge, 115-8.2002.

[20] Qin P, Agerbo E, Westergard-Nielsen N, Eriksson T, Mortensen PB Gender differences in risk factors for suicide in Denmark. Br J Psychiatry 2000; 177: 546-50. 11104395 10.1192/bjp.177.6.546

[21] Blakely TA, Collings SCD, Atkinson J Unemployment and suicide: Evidence for a causal association? J Epidemiol Community Health 2003; 57: 594-600. 12883065 10.1136/jech.57.8.594 PMC1732539

[22] Hemenway D, Solnick SJ, Colditz GA Smoking and suicide among nurses. Am J Public Health 1993; 83: 249-51. 8427332 10.2105/ajph.83.2.249 PMC1694571

[23] Miller M, Hemenway D, Rimm E Cigarettes and suicide: a prospective study of 50000 men. Am J Public Health 2000; 90: 768-73. 10800427 10.2105/ajph.90.5.768 PMC1446219

[24] Caces F, Harford T Time series analysis of alcohol consumption and suicide mortality in the United States, 1934-1987. J Stud Alcohol 1998; 59: 455-61. 9647428 10.15288/jsa.1998.59.455

[25] Brown DR, Blanton CJ Physical activity, sports participation, and suicidal behavior among college students. Med Sci Sports Exerc 2002;34:1087-96. 12131246 10.1097/00005768-200207000-00006

[26] Sobue T, Yamamoto S, Watanabe S Smoking and drinking habits among the JPHC Study participants at baseline survey. Japan Public Health Center-based Prospective Study on Cancer and Cardiovascular Diseases. J Epidemiol 2001; 11 (Suppl): S44-S56. 11763139 10.2188/jea.11.6sup_44 PMC11858363

[27] Mino Y, Aoyama H, Froom J Depressive disorders in Japanese primary care patients. Fam Pract 1994; 11: 363-7. 7895962 10.1093/fampra/11.4.363

[28] Kageyama T, Kabuto M, Nitta H, Kurokawa Y, Taira K, Suzuki S, Prevalence of use of medically prescribed hypnotics among adult Japanese women in urban residential areas. Psychiatry Clin Neurosci 1998; 52: 69-74. 9682936 10.1111/j.1440-1819.1998.tb00975.x

